# Physical Activity and Risk of Atrial Fibrillation: A Nationwide Cohort Study in General Population

**DOI:** 10.1038/s41598-019-49686-w

**Published:** 2019-09-13

**Authors:** Moo-Nyun Jin, Pil-Sung Yang, Changho Song, Hee Tae Yu, Tae-Hoon Kim, Jae-Sun Uhm, Jung-Hoon Sung, Hui-Nam Pak, Moon-Hyoung Lee, Boyoung Joung

**Affiliations:** 1 Division of Cardiology, Severance Cardiovascular Hospital, Yonsei University College of Medicine, Seoul, Republic of Korea; 2 Division of Cardiology, CHA Bundang Medical Center, CHA University, Seongnam, Republic of Korea

**Keywords:** Lifestyle modification, Atrial fibrillation

## Abstract

Although exercise prevents cardiovascular disease and mortality, vigorous exercise and endurance athletics can cause atrial fibrillation (AF). However, no large cohort study has assessed the relationship between physical activity and AF in the general population. We assessed the effect of physical activity at different energy expenditures on the incidence of AF. We studied 501,690 individuals without pre-existing AF (mean age, 47.6 ± 14.3 years; 250,664 women [50.0%]) included in the Korean National Health Insurance Service database. The physical activity level was assessed using a standardized self-reported questionnaire at baseline. During a median follow-up of 4 years, 3,443 participants (1,432 women [41.6%]) developed AF. The overall incidence of AF at follow-up was 1.79 per 1,000 person-years. The subjects who met the recommended physical activity level (500–1,000 metabolic equivalent task [MET] minutes/week) had a 12% decreased AF risk (adjusted hazard ratio [HR]: 0.88, 95% confidence interval [CI]: 0.80–0.97), but not the insufficiently (1–500 MET-minutes/week; HR: 0.94, 95% CI: 0.86–1.03) and highly active subjects (≥1,000 MET-minutes/week; HR: 0.93, 95% CI: 0.85–1.03). The recommended minimum key target range of physical activity level was associated with the maximum benefit for reduced AF risk in the general population. The dose-response relationship between physical activity level and AF risk showed a U-shaped pattern. Although exceeding the key target range attenuated this benefit, it did not increase the AF risk beyond that during inactivity.

## Introduction

Atrial fibrillation (AF) is the most common cardiac arrhythmia worldwide and can increase the risk of stroke, heart failure, dementia, and mortality^1–3^. The lifetime AF risk is one in four individuals aged >40 years^4^. Its prevalence will increase rapidly with the aging society, and the public health burden imposed by AF and AF-related morbidity is enormous. The population attributable risk of AF for lifestyle based risk factors is more than half^5^. Regular physical activity has favorable effects on cardiovascular risk factors, including significant reductions in cardiovascular morbidity and mortality for those meeting the recommended level.

The potential benefit of regular physical activity in reducing AF risk may be considerable. However, the relationship between physical activity level and AF risk remains unclear; the reported relationship varied among studies. Moderate physical activity is related to reduced AF risk, while high-intensity or endurance exercise is associated with increased AF risk in younger athletes and middle-aged men^6–10^. Thus, the effect of different physical activity levels on the incidence of AF varies. However, no eligible studies have addressed whether the public health guideline-recommended physical activity level can positively affect the AF risk. Moreover, the optimal target range for AF prevention remains undefined.

In this study, we evaluated the association of physical activity level and new-onset AF in a community-based database in Korea, which includes >500,000 individuals who underwent health examinations. Moreover, we assessed the effect of different physical activity levels on the incidence of AF and aimed to suggest the optimal target range of physical activity for AF prevention.

## Methods

### Data source

This study was based on the Korean National Health Insurance Service (NHIS) sample cohort (from 2002 to 2013) database released in 2015. The NHIS manages all Korean health service databases as a single-insurer system. The sample cohort was extracted via probability sampling from all beneficiaries of the NHIS and National Medical Aid depending on the entire national cohort information. The sample cohort database consisted of the dataset of the sociodemographic information of the beneficiaries; medical claims dataset, including information on the diagnosis based on the 10th revision of the International Classification of Disease (ICD-10) codes, treatment, and admission; and National Health Screening dataset. The National Health Screening dataset comprised a health check-up database conducted for the entire Korean population from 2002 to 2013 by the National Health Insurance Corporation. The National Health Screening Program included chest X-ray, blood test, physical examination, anthropometric measurement, and use of a questionnaire related to physical activity. Approximately 70% of the entire cohort underwent a national health screening program^11^. The death registration database of the Korea National Statistical Office, including the date and cause of the death, was linked with the NHIS cohort database. Every population in the sample cohort was linked by the Korean social security numbers, and all social security numbers were deleted after constructing the cohort by providing serial numbers to prevent leakage of personal information. This study was approved by the Institutional Review Board (IRB) of Yonsei University College of Medicine in Seoul, Republic of Korea. The IRB waived the requirement to obtain informed consent, and this study was conducted in accordance with the tenets of the Declaration of Helsinki.

### Study population

This study investigated adults without AF aged >18 years receiving the National Health Screening Program in 2009 among the total population included in the sample cohort. Data of 501,690 participants were available for analyses. To include only newly diagnosed AF, we selected all patients with AF not diagnosed during the 3-year blanking period from January 2002 to December 2004. AF was defined according to ICD-10 code I48 and presence of one hospitalization or two outpatient visits. To evaluate the accuracy of our definition of AF, we conducted a validation study in two hospitals with 628 randomly selected patients with ICD-10 code I48. Their electrocardiograms were reviewed by two physicians. The patients were ascertained to have AF, with a positive predictive value of 94.1%^12,13^. The participants were followed up at the time of incident AF, disqualification by the NHIS (mortality or immigration), or end of the study (December 31, 2013). The details regarding profile of this cohort and definition of AF have been well described in previously published articles^11–15^.

### Physical activity level assessment

The National Health Screening Program included health examination and interview. The health interview included a self-administered standardized questionnaire for ascertaining the physical activity status^16,17^. Each participant was asked to report the frequency of physical activity weekly in three categories: vigorous (20-minute increments), moderate (30-minute increments), and walking (30-minute increments). Vigorous-intensity physical activity was defined as intense exercise that causes severe shortness of breath such as running. Moderate-intensity physical activity was defined as exercise that causes mild shortness of breath such as brisk walking and bicycling at a usual speed. Walking was defined as usual-pace walking for at least 10 minutes at a time, including transportation. Each type of activity was assigned a metabolic equivalent task (MET) score based on the energy cost^18,19^. We computed the energy expended in each activity by multiplying the associated MET score by the minutes on the activity and summed the energy expended across the moderate- to vigorous-intensity activities to estimate the total energy expenditure per week.

The participants were stratified on the basis of their weekly physical activity levels as follows: (1) inactive group: no leisure-time physical activity (LTPA) beyond basic movements; (2) insufficiently active group: having less than the key guideline target range (1–500 MET-minutes/week); (3) active group: meeting the key guideline target range (500–1,000 MET-minutes/week); and (4) highly active group: exceeding the key guideline target range (>1,000 MET-minutes/week)^20,21^.

### Statistical analysis

Descriptive statistics were used to characterize the baseline characteristics and comorbidities. The values were presented as means ± standard deviations or as percentages, as appropriate. Cox proportional hazard regression was used to assess the association between AF and LTPA. Models for clinical variable adjustments were used to assess the association. A multivariable model was adjusted for age, sex, body mass index (BMI), heart failure, hypertension, diabetes, previous myocardial infarction (MI), prior stroke or transient ischemic attack, chronic kidney disease (CKD), smoking, and alcohol drinking. To assess the dose-response relationship LTPA and mortality, we used restricted cubic spline model. We further examined the physical activity levels by separating moderate-intensity and vigorous-intensity activities and creating mutually adjusted models. We also stratified them by potential effect modifiers of age, sex, BMI, waist circumflex, heart failure, hypertension, blood pressure, diabetes, CKD, previous MI, dyslipidemia, smoking, and alcohol drinking. A p-value of <0.05 was considered statistically significant. Statistical analysis was performed using the Statistical Package for Social Sciences version 23.0 (SPSS Inc., Chicago, IL, USA) and R version 3.4.4 (R Foundation for Statistical Computing, Vienna, Austria).

## Results

### Baseline characteristics

In total, 501,690 participants (50% women) with a mean age of 47.6 ± 14.3 years were included in the analyses. The baseline characteristics of the study participants stratified according to their LTPA are compared in Table 1. With regard to physical activity level, 25.0% of the participants were inactive; 31.4% performed insufficiently active LTPA (1–500 MET-minutes/week); 26.5% performed active LTPA (500–1,000 MET-minutes/week); and 17.2% performed highly active LTPA (>1,000 MET-minutes/week).

**Table 1:** Baseline characteristics stratified by the leisure-time physical activity level.

Characteristic	ALL	Physical activity level, MET min/week				
		None	1 to <500	500 to <1000	≥1000	P for trend
Participants	501,690	125,259 (25.0%)	157,515 (31.4%)	132,823 (26.5%)	86,093 (17.2%)	
Age	47.6 ± 14.3	50.4 ± 14.6	45.7 ± 13.9	46.5 ± 14.3	48.7 ± 13.9	<0.001
Female	250,664 (50.0%)	69,307 (55.3%)	81,601 (51.8%)	62,427(47.0%)	15,537 (42.6%)	<0.001
BMI (kg/m^2^)	23.7 ± 3.3	23.7 ± 3.4	23.5 ± 3.4	23.7 ± 3.3	24.0 ± 3.1	<0.001
Waist circumflex	80.0 ± 9.4	80.2 ± 9.5	79.6 ± 9.5	80.0 ± 9.3	80.6 ± 8.9	<0.001
Heart failure	10,937 (2.2%)	3,798 (3.0%)	2,883 (1.8%)	2,458 (1.9%)	1,798 (2.1%)	<0.001
Hypertension	107,925 (21.5%)	31,072 (24.8%)	29,056 (18.4%)	26,872 (20.2%)	20,925 (24.3%)	0.001
Diabetes	31,396 (6.3%)	8,590 (6.9%)	8,129 (5.2%)	8,037 (6.1%)	6,640 (7.7%)	<0.001
Previous MI	4,555 (0.9%)	1,416 (1.1%)	1,177 (0.7%)	1,104 (0.8%)	858 (1.0%)	0.001
History of stroke/TIA	18,461 (3.7%)	5,986 (4.8%)	4,821 (3.1%)	4,404 (3.3%)	3,250 (3.8%)	<0.001
Chronic kidney disease	29,265 (5.8%)	8,496 (6.8%)	8,104 (5.1%)	7,470 (5.6%)	5,195 (6.0%)	<0.001
Dyslipidemia	143,497 (28.6%)	38,329 (31.0%)	42,371 (26.9%)	36,659 (27.6%)	26,138 (30.4%)	<0.001
COPD	11,453 (2.3%)	3,841 (3.1%)	3,074 (2.0%)	2,639 (2.0%)	1,899 (2.2%)	<0.001
Cancer	33,396 (6.7%)	9,065 (7.2%)	9,331 (5.9%)	8,421 (6.3%)	6,579 (7.6%)	0.003
Smoker (Ex or current)	188,985 (37.7%)	40,409 (32.3%)	58,690 (37.3%)	54,192 (40.8%)	35,694 (41.5%)	<0.001
Alcohol drinker ( ≥ 1time/week)	236,772 (47.2%)	45,669 (36.5%)	77,592 (49.3%)	69,121 (52.0%)	44,390 (51.6%)	<0.001
Systolic BP (mmHg)	122.3 ± 15.3	123.0 ± 15.9	121.3 ± 15.1	122.1 ± 15.1	123.1 ± 15.0	<0.001
Diastolic BP (mmHg)	76.1 ± 10.2	76.4 ± 10.4	75.7 ± 10.2	76.0 ± 10.1	76.5 ± 10.1	<0.001
Fasting glucose (mg/dl)	97.9 ± 24.8	98.9 ± 26.7	96.9 ± 23.5	97.5 ± 24.2	98.7 ± 25.1	<0.001
Total cholesterol (mg/dl)	194.9 ± 37.3	196.1 ± 38.1	194.7 ± 37.1	194.3 ± 36.9	194.7 ± 37.1	<0.001
Triglyceride	132.0 ± 93.9	136.0 ± 95.5	131.8 ± 93.6	130.7 ± 94.1	128.9 ± 91.5	<0.001
LDL (mg/dl)	113.7 ± 37.2	114.7 ± 38.9	113.4 ± 36.5	113.1 ± 36.6	113.6 ± 37.0	<0.001
HDL (mg/dl)	56.4 ± 27.2	56.7 ± 33.5	56.2 ± 25.3	56.3 ± 24.7	56.5 ± 24.0	<0.001

Values are expressed as n (%) or means ± standard deviations.

BMI: body mass index, BP: blood pressure, COPD: chronic obstructive pulmonary disease, HDL: high-density lipoprotein, LDL: low-density lipoprotein, MET: metabolic equivalent task, MI: myocardial infarction, TIA: transient ischemic attack.

### Physical activity and AF

**Figure 1:**
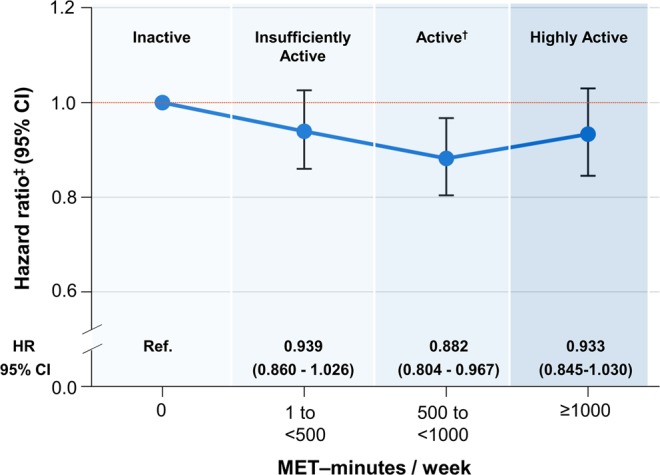
Multivariable adjusted relative risk of atrial fibrillation according to physical activity level as classified in the Physical Activity Guidelines. ^†^Minimum key guidelines target range in Physical Activity Guidelines for Americans by the U.S. Department of Health and Human Services (2018): Doing the equivalent of 500 to 1,000 MET-minutes a week of physical activity. ^‡^Multivariable adjusted hazard ratio (HR): age, sex, BMI, HF, HTN, DM, Previous MI, Stroke or TIA, CKD, Smoking, and Alcohol.

During a median follow-up of 4 years, 3,443 participants (1,432 women [41.6%]) developed AF. The overall incidence of AF at follow-up was 1.79 per 1,000 person-years. The cumulative incidence of AF according to physical activity levels presented in Supplementary Fig. 1. When stratified by physical activity level, the incidence was 2.27, 1.52, 1.56, and 1.91 per 1,000 person-years in the inactive, insufficiently active, active, and highly active groups, respectively (Table 2). There was a U-shaped relationship between physical activity level and AF risk (Fig. 1). The active group (500–1,000 MET-minutes/week) had a 12% lower risk of AF (adjusted hazard ratio [HR]: 0.88, 95% confidence interval [CI]: 0.80–0.97) than the inactive group. However, the insufficiently active group (1–500 MET-minutes/week; HR: 0.94, 95% CI: 0.86–1.03) and highly active group (≥1,000 MET-minutes/week; HR: 0.93, 95% CI: 0.85–1.03) had a modest but nonsignificant 6% and 7% lower risk of AF, respectively (Table 2).

**Table 2:** Risk of atrial fibrillation in relation to the total leisure-time physical activity.

	Physical activity level, MET min/week			
	None	1 to <500	500 to <1000	≥1000
Incidence (%)	1,084 (0.87%)	925 (0.59%)	796 (0.60%)	638 (0.74%)
Incidence (/1000 person-years)	2.27	1.52	1.56	1.91
Hazard ratio (95% confidence interval),				
Age and sex adjusted	1.00 (ref.)	0.94 (0.86–1.02)	0.88 (0.80–0.96)	0.94 (0.85–1.04)
P value		0.145	0.005	0.211
Fully adjusted^†^	1.00 (ref.)	0.94 (0.86–1.03)	0.88 (0.80–0.97)	0.93 (0.85–1.03)
P value		0.165	0.007	0.172

^†^The model was adjusted for age, sex, body mass index, heart failure, hypertension, diabetes, previous myocardial infarction, prior stroke or transient ischemic attack, chronic kidney disease, smoking, and alcohol drinking.

MET: metabolic equivalent task.

Cox models with cubic splines showed a shape of the dose-response curve for the relationship between physical activity and risk of AF (P non-linearity = 0.08, Supplementary Fig. 2). Physical activity at volumes of about 400–600 MET-minutes/week was associated with reduction for risk of AF. Physical activity volume over 1000 MET-minutes/week was not related with risk of AF, however, its relative risk was not higher than inactivity.

### Exercise intensity and AF

We employed separate moderate-intensity and vigorous-intensity categories ranging from 0 to >1,000 MET-minutes/week, and the analysis models were mutually adjusted for both LTPA intensities (Table 3). We found an AF risk reduction in moderate-intensity LTPA (500–1,000 MET-minutes/week: HR: 0.90, 95% CI: 0.82–0.98). However, there was no reduction in the incidence of AF in vigorous-intensity LTPA. Even among the participants meeting the guideline-recommended LTPA level, the risk of AF significantly decreased after performing moderate-intensity LTPA; the risk did not decrease after performing vigorous-intensity LTPA.

**Table 3:** Risk of atrial fibrillation according to the intensity of physical activity.

Hazard ratio (95% confidence interval)	Physical activity level, MET min/week			
	None	1 to <500	500 to <1000	≥1000
Moderate				
Age and gender adjusted	1.00 (ref.)	0.96 (0.89–1.05)	0.89 (0.82–0.98)	0.88 (0.76–1.02)
P value		0.383	0.014	0.095
Fully adjusted^†^	1.00 (ref.)	0.96 (0.89–1.05)	0.90 (0.82–0.98)	0.88 (0.76–1.02)
P value		0.393	0.018	0.082
Vigorous				
Age and gender adjusted	1.00 (ref.)	0.98 (0.89–1.07)	1.06 (0.93–1.21)	1.08 (0.93–1.25)
P value		0.629	0.359	0.334
Fully adjusted^†^	1.00 (ref.)	0.99 (0.90–1.08)	1.05 (0.93–1.20)	1.07 (0.92–1.23)
P value		0.754	0.434	0.400

^†^The model was adjusted for age, sex, body mass index, heart failure, hypertension, diabetes, previous myocardial infarction, prior stroke or transient ischemic attack, chronic kidney disease, smoking, and alcohol drinking and mutually adjusted for both moderate- and vigorous-intensity activities.

MET: metabolic equivalent task.

**Figure 2:**
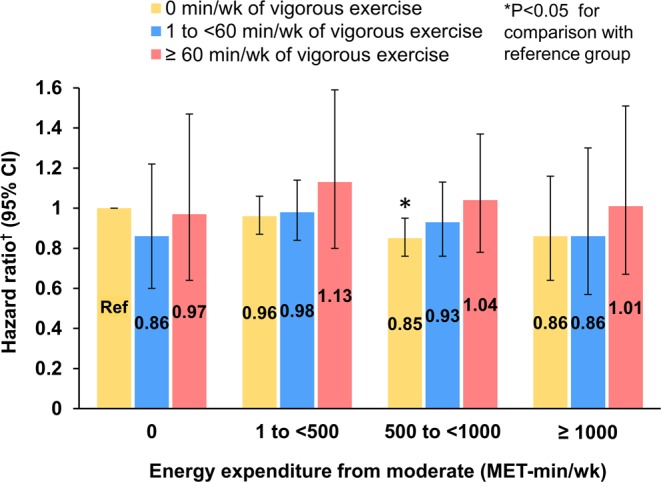
Joint association of moderate-intensity and vigorous-intensity physical activities with the adjusted relative risk of atrial fibrillation. ^†^The multivariable adjusted hazard ratio was adjusted for age, sex, body mass index, heart failure, hypertension, diabetes, previous myocardial infarction, prior stroke or transient ischemic attack, chronic kidney disease, smoking, and alcohol drinking and mutually adjusted for both moderate- and vigorous-intensity activities.

The participants who engaged in both moderate- and vigorous-intensity activities had no additional reduction in the AF risk compared with those who performed moderate-intensity activity alone. The adjusted relative risk of AF of the participants who had vigorous-intensity LTPA was insignificantly higher than that of those who had moderate-intensity LTPA (Fig. 2).

### Subgroup analysis

The adjusted AF risk according to the total physical activity level in each subgroup is presented in Table 4. The association between physical activity level and AF risk was similar across the subgroups; the maximum benefit for AF risk reduction was associated with meeting the key target range of physical activity, with interactions indicating greater benefits in patients with blood pressures below 130/80 mmHg and without central obesity.

**Table 4:** Association between increasing physical activity level and risk of atrial fibrillation according to subgroups.

Characteristic	Fully adjusted hazard ratio^†^ (95% confidence interval)				
	Physical activity level, MET*min/week				
	None	1 to <500	500 to <1000	≥1000	P for interaction
Age					0.210
<50	1.00 (ref.)	0.93 (0.74–1.17)	0.81 (0.63–1.04)	0.81 (0.61–1.08)	
50 to 64	1.00 (ref.)	0.98 (0.84–1.15)	0.94 (0.80–1.11)	1.05 (0.89–1.23)	
≥65	1.00 (ref.)	0.92 (0.82–1.04)	0.85 (0.78–0.97)	0.85 (0.74–0.98)	
Sex					0.223
Male	1.00 (ref.)	0.92 (0.81–1.03)	0.92 (0.81–1.03)	0.96 (0.85–1.09)	
Female	1.00 (ref.)	0.97 (0.85–1.10)	0.83 (0.72–0.96)	0.89 (0.75–1.05)	
BMI					0.084
<25	1.00 (ref.)	0.99 (0.88–1.11)	0.88 (0.78–0.99)	0.89 (0.78–1.02)	
≥25	1.00 (ref.)	0.88 (0.77–1.02)	0.90 (0.78–1.04)	1.02 (0.88–1.19)	
Central obesity by waist					0.033
Yes	1.00 (ref.)	0.95 (0.83–1.08)	0.87 (0.76–1.003)	1.05 (0.90–1.22)	
No	1.00 (ref.)	0.94 (0.83–1.06)	0.89 (0.79–1.008)	0.87 (0.76–0.99)	
Heart failure					0.622
Yes	1.00 (ref.)	1.01 (0.79–1.28)	0.88 (0.67–1.15)	0.95 (0.71–1.27)	
No	1.00 (ref.)	0.93 (0.85–1.02)	0.88 (0.80–0.97)	0.92 (0.83–1.03)	
Hypertension					0.193
Yes	1.00 (ref.)	0.94 (0.84–1.06)	0.85 (0.75–0.96)	0.87 (0.76–0.99)	
No		0.95 (0.82–1.09)	0.93 (0.80–1.07)	1.00 (0.86–1.17)	
High BP (>130/80 mmHg)					0.028
Yes	1.00 (ref.)	0.98 (0.88–1.10)	0.93 (0.83–1.05)	1.01 (0.89–1.14)	
No	1.00 (ref.)	0.87 (0.75–1.01)	0.80 (0.68–0.93)	0.81 (0.69–0.96)	
Diabetes					0.857
Yes	1.00 (ref.)	1.03 (0.83–1.28)	0.87 (0.69–1.09)	0.92 (0.73–1.17)	
No	1.00 (ref.)	0.92 (0.84–1.02)	0.88 (0.80–0.98)	0.93 (0.84–1.04)	
Chronic kidney disease					0.856
Yes	1.00 (ref.)	1.05 (0.86–1.28)	0.83 (0.66–1.04)	0.98 (0.77–1.25)	
No	1.00 (ref.)	0.92 (0.83–1.01)	0.89 (0.80–0.98)	0.92 (0.82–1.02)	
Previous MI					0.795
Yes	1.00 (ref.)	0.98 (0.63–1.53)	0.67 (0.40–1.12)	0.93 (0.57–1.54)	
No	1.00 (ref.)	0.94 (0.86–1.03)	0.89 (0.81–0.98)	0.93 (0.84–1.03)	
Dyslipidemia					0.752
Yes	1.00 (ref.)	0.93 (0.81–1.05)	0.86 (0.75–0.98)	0.94 (0.81–1.08)	
No	1.00 (ref.)	0.96 (0.85–1.08)	0.91 (0.80–1.03)	0.93 (0.81–1.06)	
CHDSV score^‡^					
0	1.00 (ref.)	0.96 (0.82–1.11)	0.95 (0.81–1.11)	1.0 (0.84–1.18)	0.216
≥1	1.00 (ref.)	0.94 (0.84–1.05)	0.84 (0.75–0.95)	0.88 (0.78–1.00)	
1~2	1.00 (ref.)	0.94 (0.83–1.07)	0.84 (0.74–0.96)	0.89 (0.77–1.02)	
≥3	1.00 (ref.)	0.93 (0.73–1.19)	0.84 (0.65–1.09)	0.85 (0.63–1.13)	
Alcohol (≥1time/week)					0.078
Drinker	1.00 (ref.)	0.94 (0.80–1.10)	0.95 (0.81–1.11)	1.03 (0.87–1.21)	
Non-drinker	1.00 (ref.)	0.95 (0.85–1.06)	0.85 (0.76–0.95)	0.87 (0.76–0.99)	
Smoking (former + current)					0.888
Smoker	1.00 (ref.)	0.99 (0.85–1.15)	0.91 (0.79–1.06)	0.93 (0.79–1.09)	
Never	1.00 (ref.)	0.91 (0.82–1.02)	0.86 (0.77–0.97)	0.94 (0.83–1.07)	

^†^The model was adjusted for age, sex, BMI, heart failure, hypertension, diabetes, previous MI, prior stroke or TIA, chronic kidney disease, smoking, and alcohol drinking and mutually adjusted for both moderate- and vigorous-intensity activities.

^‡^CHDSV score assigns 1 point for heart failure, hypertension, diabetes, prior stroke or TIA, and vascular disease.

BMI: body mass index, BP: blood pressure, MET: metabolic equivalent task, MI: myocardial infarction, TIA: transient ischemic attack.

## Discussion

The present study investigated the association between LTPA and development of AF in a nationwide general population. We assessed the effect of LTPA at different energy expenditures as indicated in public health guidelines for physical activity^20,21^. The key target range of physical activity (500–1,000 MET-minutes/week) was associated with the largest benefit for reduced AF risk in the general population, whereas insufficient activity (1–500 MET-minutes/week) and high activity (>1,000 MET-minutes/week) attenuated the benefit for AF risk reduction. Our analysis found an incremental reduction in incident AF after moderate-intensity physical activity; higher-intensity physical activity had no beneficial effect. The dose-response relationship between physical activity level and AF risk appeared to follow a U-shaped pattern.

### Physical activity and AF

The health benefits of regular physical activity have been well described, including significant reductions in cardiovascular mortality and morbidity for those meeting the recommended key target range. Furthermore, an additional benefit occurred with increasing physical activity level. At the minimum recommended physical activity level, the risk of mortality, coronary heart disease, and heart failure decreased; at two times the minimum recommended level, the risk decreased further^22,23^.

In this nationwide population-based large cohort study, the subjects who met the key minimum recommended target range by performing moderate- or vigorous-intensity LTPA had a 12% lower AF risk than the inactive subjects. Those exceeding the key minimum recommended target range had no significant risk reduction. Our findings are consistent with those of previous studies supporting that moderate but not vigorous physical activity decreases the AF risk. However, a higher activity level did not significantly increase the risk of AF compared with inactivity. The subjects who engaged in highly active LTPA did not appear to be at an increased risk of AF compared with those who had no LTPA. A meta-analysis including 18 cohort studies and one cross-sectional study reported that the dose-relationship between physical activity and AF had a J-shaped pattern, and a physical activity exceeding 1,200 MET-minutes/week was not associated with AF risk^24^. Some previous studies reported that increasing physical activity was probably associated with an increased risk of AF in men and a decreased risk in women^25,26^. However, our study did not find a gender-specific increased risk of AF with high volume physical activity. We believe the conflict results from different study populations. Previous studies reporting increased risk with higher physical activity level were mainly conducted among middle aged men or endurance athletes^7,27,28^, whereas our analyses conducted from general population with broad age. Zhu *et al*.^25^ meta-analysis reported that male individuals with intensive physical activity had a slightly higher risk of developing AF, but there was a significantly reduced risk of incident AF in female. Interestingly, the meta-analysis showed that increasing physical activity was probably associated with an increased risk of AF in middle- or young-aged men. In addition, the meta-analysis included only western countries data. The pattern of risk for AF with increasing physical activity level vary according to study characteristics. Therefore, self-reported physical activity, widely varying categorizing of the activity, vary in definition of AF and different cultural patterns of sport seem to limit the comparative interpretation of existing studies^29,30^.

Interestingly, there was no difference in the cumulative incidence of AF between highly active and inactive groups in men (Supplementary Fig. 1,C), whereas highly active group had lower cumulative incidence of AF than in inactive group in women (Supplementary Fig. 1,D). It may support the hypothesis which was exercise-induced enhancement of parasympathetic tone dominate in men^7^. Our findings indicate the uncertainty of the nature of the relationship at higher physical activity levels and the complex role of physical activity in modifying the risk of AF.

There are several mechanisms underlying the complex association between physical activity and AF. Physical activity can potentially reduce the risk of AF through beneficial effects on cardiovascular risk factors. Conversely, long-term high exercise levels may lead to remodeling of the heart and alteration of the autonomic nervous system^31^.

### Exercise intensity and AF

The current public health guideline did not specifically consider the relative value of different physical activity levels such as moderate- and vigorous-intensity physical activities^32^. In the separate intensity model, the AF risk significantly decreased after moderate-intensity LTPA but not after vigorous-intensity LTPA. A small number of studies have reported the AF risk according to the intensity of physical activity. The relationship between exercise intensity and AF risk suggests a curvilinear response with diminishing benefit or even a risk with the most intense exercise^33^. Mozaffarian *et al*. reported that a significant reduction in the AF risk could be observed with moderate- but not high-intensity exercise^6^. Similarly, a Norwegian community-based cohort study reported that a significant reduction in the AF risk could be observed with moderate-intensity activities such as walking and cycling, but not higher-intensity activities^30^. We believe our data are of interest because they expand our understanding of the complex role of physical activity in modifying the risk of AF in the general population. Moderate physical activity may reduce the risk of AF via modified cardiovascular risk factors, e.g., by improving weight, blood pressure, inflammation, glucose level, and lipid control^34–36^.

However, this favorable effect of moderate physical activity on AF may be mitigated by chronic exposure to vigorous physical activity, which generates structural and functional cardiac adaptations that hinder the reduction of the risk of AF. The possible mechanism of the increased AF risk in subjects exposed to vigorous physical activity may be higher inflammation and oxidative stress^37^, increased atrial ectopic burden^38^, vagal enhancement^39^, and atrial dilatation and fibrosis^40^. These mechanisms are widely documented in long-endurance training and could counteract the beneficial effect of moderate physical activity on the AF risk^41^. Further studies designed to measure physical activity exposure in terms of energy expenditure are needed to clarify the relationship between different intensities of physical activity and AF risk.

A major strength of this study is its large sample size with a broad age range from a representative nationwide cohort, and that the uniform physical activity categories used matched the levels described in the current public guidelines. In addition, limited data are available to evaluate the AF in relation to LTPA in Asian population. To the best of our knowledge, this was the largest Asian population-based study investigating the association between physical activity and AF. However, some limitations must also be considered.

### Study limitations

The limitations of this study include the reliance on the self-reported physical activity, which was reported at a single time point. This may cause inaccurate measurements of self-reported LTPA habits, and the conditions at the time of questionnaire completion may not represent the actual LTPA conditions throughout life. Further, behavioral changes during the follow-up period could not be assessed in our study. Nevertheless, self-report questionnaires may provide a reliable approximation of PA at a population level^42^, and validity of IPAQ based self-reported physical activity questionnaire has been confirmed in several studies and has yielded valuable results to date^43^. In addition, although the South Korean society has changed radically and has become more westernized, caution is needed for generalization of our results to western countries owing to racial, ethnic, and geographic restrictions. This study is potentially susceptible to errors arising from coding inaccuracies and cases of asymptomatic paroxysmal AF may have been missed because the research used administrative database. To minimize this problem, we applied the definition that we already validated in previous studies that used the Korean NHIS sample cohort and the diagnostic reliability of cohort data was high^12–15^. The validity of hazard ratios depend on the completeness of follow-up because the analysis was performed retrospectively. The NHIS in Korea is a universal coverage and single-insurer system, we believe that very small participants were lost to follow-up. Unfortunately, we were unable to adjust for variables that were not included in the cohort, such as occupational physical activity, echocardiogram and obstructive sleep apnea data.

## Conclusions

Meeting the recommended key target range of physical activity was associated with the maximum benefit for AF risk reduction in the general population. The dose-response relationship between physical activity level and AF risk showed a U-shaped pattern. Although exceeding the key target range attenuated this benefit, it did not increase the risk of AF beyond that during inactivity. Our findings support the current public guideline-recommended 150-minute/week moderate-intensity physical activity for achieving health benefits. Although vigorous LTPA should not be discouraged for those who want a higher intensity of activity, our results indicate that moderate-intensity exercise is more recommended to achieve substantial health benefits for AF risk reduction.

## Supplementary information

Supplementary Information
