# Supplementary material for: Physical Activity and Risk of Atrial Fibrillation: A Nationwide Cohort Study in General Population
Source: Sci Rep. 2019 Sep 13;9:13270. doi: 10.1038/s41598-019-49686-w (PMC6744571; doi:10.1038/s41598-019-49686-w)

Supplementary Information

Physical Activity and Risk of Atrial Fibrillation: A Nationwide Cohort Study in General Population

Moo-Nyun Jin, MD[1]; Pil-Sung Yang, MD[2]; Changho Song, MD[1]; Hee Tae Yu, MD, PhD[1]; Tae-Hoon Kim, MD[1]; Jae-Sun Uhm, MD, PhD[1]; Jung-Hoon Sung, MD, PhD[2]; Hui-Nam Pak, MD, PhD[1]; Moon-Hyoung Lee, MD, PhD[1]; and Boyoung Joung, MD, PhD[1]

[1]*Division of Cardiology, Severance Cardiovascular Hospital, Yonsei University College of Medicine, Seoul, Republic of Korea*

[2]*Division of Cardiology, CHA Bundang Medical Center, CHA University, Seongnam, Republic of Korea*

Supplementary Figure 1. Cumulative incidence of atrial fibrillation by a Kaplan-Meier curves. (A) Comparison in different physical activity levels in overall study population; (B) Comparison between men and women; (C) Comparison in different physical activity levels in men; (D) Comparison in different physical activity levels in women

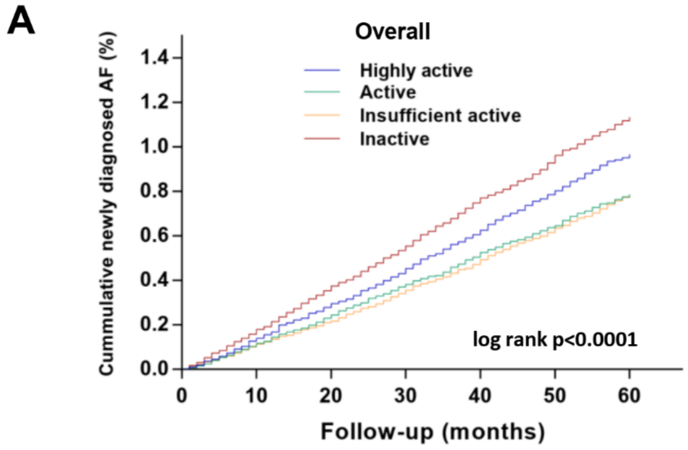

No. at risk

Inactive	125259	124977	115841	102652	85103	50217	49830
Insufficient active	157515	157323	145763	129729	108477	65751	65460
Active	132823	132657	122667	109072	90749	54279	54056
Highly active	86093	85987	79714	71364	60062	35874	35698

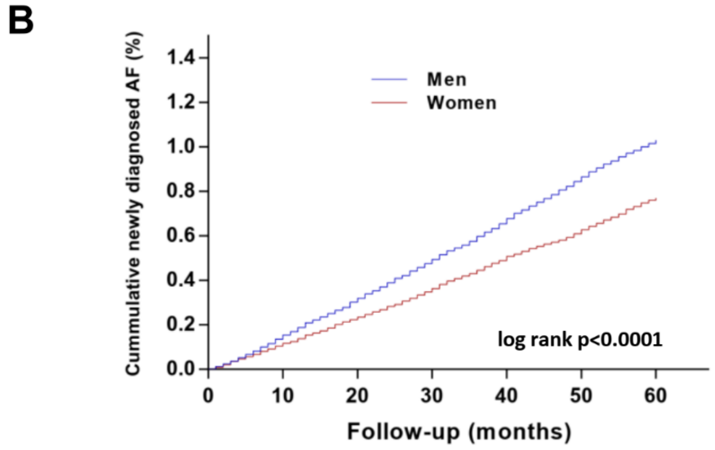

No. at risk

Men	251026	250601	232817	208530	175993	112212	111526
Women	250664	250343	231168	204287	168398	93909	93518

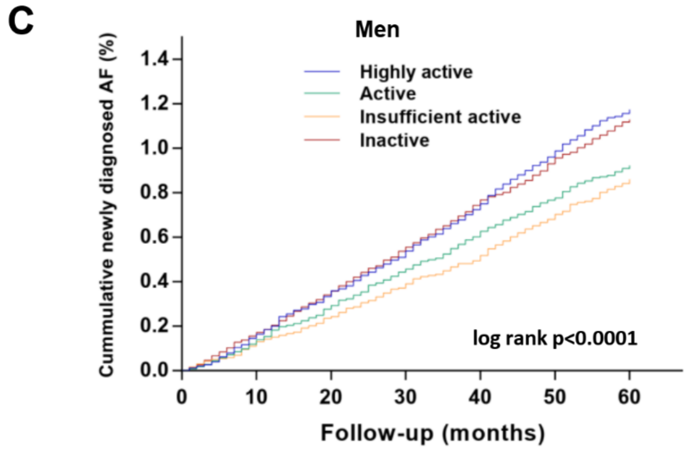

No. at risk

Inactive	85951	85752	79506	70683	59062	36993	36695
Insufficient active	45915	45859	42995	38952	33336	22155	22052
Active	70396	70293	65154	58323	49089	31281	31128
Highly active	48764	48697	45162	40572	34506	21783	21651

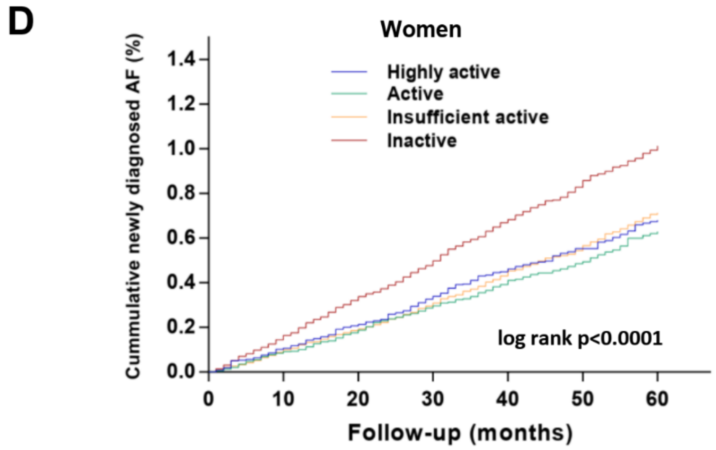

No. at risk

Inactive	69307	69174	64031	56637	46762	26243	26081
Insufficient active	81601	81515	75072	66109	54420	30577	30462
Active	62427	62364	57513	50749	41660	22998	22928
Highly active	37329	37290	34552	30792	25556	14091	14047

Supplementary Figure 2. Dose-response analyses of the association between total leisure time physical activity and risk of atrial fibrillation

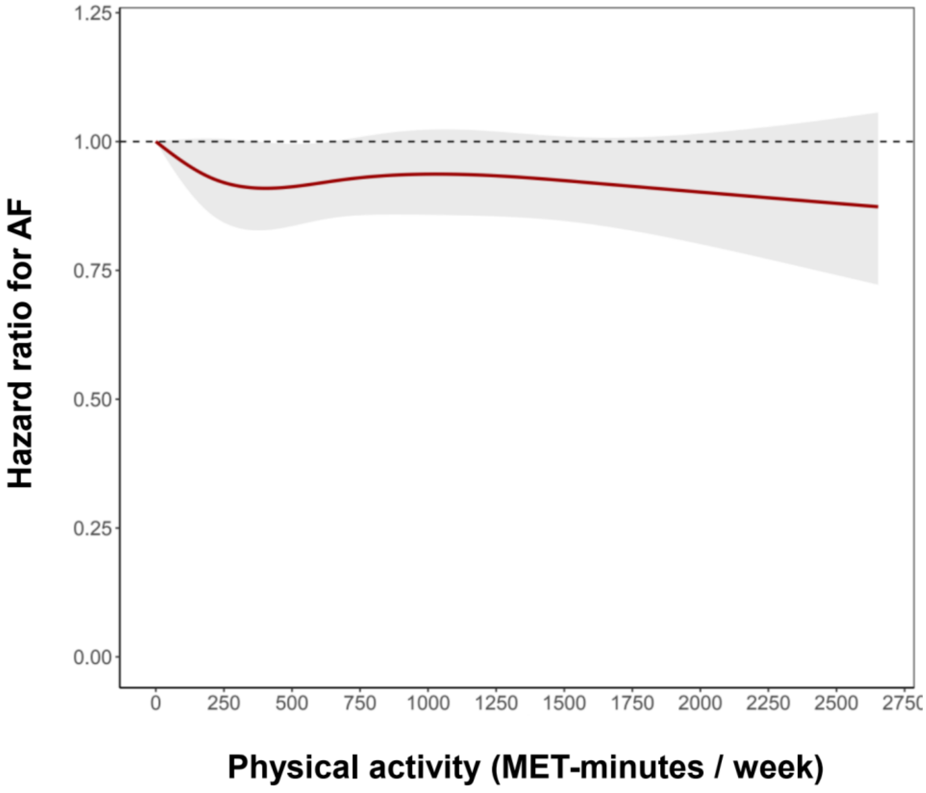

Supplement: Supplementary file 1 — Supplementary Information [file 41598_2019_49686_MOESM1_ESM.pdf]
